# Fly Me to the Immune: Immunonutrition in Rheumatic Diseases

**DOI:** 10.31138/mjr.34.1.30

**Published:** 2023-02-21

**Authors:** Maria G. Grammatikopoulou, Georgios Marakis, Konstantinos Gkiouras, Dorothea Athanatou, Maria I. Maraki, Dimitrios P. Bogdanos

**Affiliations:** 1 Immunonutrition Unit, Department of Rheumatology and Clinical Immunology, General University Hospital of Larissa, Faculty of Medicine, School of Health Sciences, University of Thessaly, Larissa, Greece,; 2 Nutrition and Food Standards Unit, Directorate of Risk Assessment and Nutrition, Hellenic Food Authority, Athens, Greece,; 3 Department of Nutrition and Dietetics, School of Health Sciences, Hellenic Mediterranean University, Crete, Greece

**Keywords:** vitamin D, dysbiosis, microbiota, anti-inflammatory diet, rheumatoid arthritis, lupus, psoriasis

## Abstract

Immunonutrition is the maintenance and induction of immune homeostasis with the use of nutritional factors, the so called, immunonutrients. Immunonutrition focuses on four “Is” representing an equal number of systemic responses with regards to: a) Immunity, b) Infection, c) Inflammation and d) Injury. Although at the early stages of the development of immunonutrition, its application was focused on malnourished patients, with a latter extension in the intensive care unit setting, today we acknowledge the great importance of immunonutrients in rheumatology. In rheumatic diseases (RDs), all the “Is” representing the four aims and targets of immunonutrition are fulfilled. Impaired Immunity is the hallmark of RDs, with both innate and adaptive immunity contributing to the development and course of each disease entity, representing distinct immunoregulation abnormalities, often paired with micronutrient deficiencies. Infections are both drivers and a frequent epiphenomenon of systemic RDs. Subclinical inflammation is propagated long before the first signs or symptoms of RDs and musculoskeletal conditions (injury) are apparent in all patients with RDs, accompanied by pain, underlying connective tissue disease and the consequent reduction in the function of musculoskeletal. Herein, the role of probiotics, curcumin, vitamins, Selenium, Zinc and n-3 fatty acids as immunonutrients is discussed.

## INTRODUCTION

In 1968, a monograph by the World Health Organization provided the first links between infection and malnutrition.^1^ The advancement of immunology thereafter increased the amount of data explaining the interaction between nutrition and immune response.^2^ Today, this inter-relationship is widely acknowledged, as any single or combined nutrient deficiency consists of a frequent trigger for a reduced immune response.^2^ Furthermore, this observation extends far beyond nutritionally deprived populations living in developed countries, and is also apparent among people in industrialised societies.^3^

## IMMUNONUTRITION

**Figure 1.**
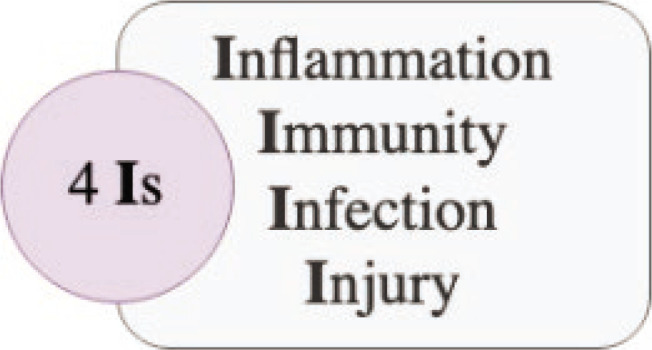
The four “**I**s” of immunonutrition according to Calder.^4^

According to Calder,^4^ the maintenance and induction of immune homeostasis with the use of nutritional factors is termed as “immunonutrition”. Successful immune responses are driven by meticulous orchestrations and the fine tuning of complex pathways and a careful provision of immune modulating substances.^5^ Immunonutrition can be applied in any situation where a dose, greater than normal, of a specific nutrient is provided, aiming to target inflammatory, metabolic, and immune pathways.^6^ Immunonutrition focuses on four “***I**s*” (**Figure 1**), representing an equal number of systemic responses with regard to a) ***I**mmunity*, b) ***I**nfection*, c) ***I**nflammation* and d) ***I**njury*.^2^ The nutrients identified as drivers of immune response in an aggregate form of a nutrition support regime are termed “immunonutrients”. The main immunonutrients include amino acids (arginine, glutamine, etc.), n-3 fatty acids, probiotics, and several antioxidants (vitamin C, carotenoids, Selenium, etc.).^7^

## IMMUNONUTRITION IN RHEUMATIC DISEASES

At the early stages of the development of immunonutrition, its application was focused on malnourished patients, with a latter extension in the intensive care unit setting. The efficacy of immunonutrients in rheumatic diseases (RDs) was not yet widely applied, despite the fact that for several RDs, including gout, the first-line treatment involved dietary interventions.

Patients with RDs often experience a variety of nutrition-related issues, including malnutrition,^8^ rheumatoid cachexia,^9,10^ overweight and obesity,^8,11,12^ gut dysbiosis,^13–16^ nutritional deficiencies^11,17–20^ and many more. Moreover, the pathophysiology of RDs induces important changes in the nutritional status of the affected populations, including hypermetabolism and increased oxidative stress.^21^ All aforementioned issues interact with immune homeostasis, increasing inflammation and disease activity. In fact, the role of chronic inflammation in vascular dysfunction and progression of atherosclerosis -which is the main cause of cardiovascular disease (CVD) in autoimmune RDs-, is increasingly acknowledged.^22^ As a result, the importance of complementary immunonutrition in rheumatology is pivotal for inducing and maintaining low levels of inflammation and disease activity, as well as for attaining the maximum health status of patients with RDs. Collectively, these findings indicate that as far as rheumatology is concerned, immunonutrition is a common issue in everyday clinical practice, instead of an occasional one, as in surgery.

In fact, in RDs, all the “***I**s*” representing the four aims and targets of immunonutrition are instantly ticked. Impaired ***I**mmunity* is the hallmark of RDs, with both innate and adaptive immunity contributing to the development and course of each disease entity, representing distinct immunoregulation abnormalities.^23–25^ Moreover, fine tuning immunometabolism can be applied to provide the desired immune response, or prevent a deleterious one.^26^ ***I**nfections* are both drivers and a frequent epiphenomenon of systemic RDs.^27–34^ Localised and/or systemic ***I**nflammation* is the core characteristic of RDs, with each disease propagating distinct stimuli that targets specific tissues, while presenting different predominant inflammatory mechanisms in each case.^35^ In fact, subclinical inflammation is propagated long before the first signs or symptoms of RDs are apparent.^36^ As for the final “***I***”, namely ***I**njury*, musculoskeletal conditions are apparent in all patients with RDs,^37–39^ accompanied by pain, underlying connective tissue disease and the consequent reduction in the function and range of motion in one or more parts of the musculoskeletal system.^40,41^ Furthermore, in RDs, all “***I**s*” are linked in a perpetual vicious circle of continuity, with each domain interacting and feeding the rest.

## HIGH-HIERARCHY EVIDENCE FOR THE EFFICACY OF IMMUNONUTRITION IN RDS

A great number of primary studies and high hierarchy evidence from evidence synthesis has evaluated the effect of immunonutrition in patients with RDs. *Α* handful of the current evidence synthesis to date, highlighting the role of immunonutrient supplementation in RDs, involves supplementation with probiotics, antioxidant compounds and vitamins.

### Vitamins

Decreased availability of serum 25-hydroxyvitamin D (25[OH]D) has been postulated to compromise immune cell synthesis of hormonal 1,25-dihydroxyvitamin D, leading to impaired innate immunity and over-exuberant inflammatory adaptive immunity.^42^ Patients with SLE,^43^ fibromyalgia,^20^ SSc,^44,45^ AS,^46^ SS,^47^ and psoriasis^48^ demonstrate low vitamin D levels, which tend to correlate inversely with disease activity. In rheumatology, vitamin D supplementation is prescribed for the prevention of glucocorticoid-induced osteoporosis, the reduction of fracture-risk,^49^ and the management of pain (mainly in fibromyalgia).^50,51^ Furthermore, vitamin D ONS can improve vitamin status, protecting against relapse and deteriorating severity in RDs, given its immunologic effects on the innate and adaptive immune system, as well as on the stability of the endothelial membrane (immunometabolism).^52–54^ In RA, ONS with vitamin D improves disease activity, erythrocyte sedimentation rate (ESR), the number of tender joints,^55^ and the rate of RA reccurence.^56^ Furthermore, in patients of European descent, a reduction in pain and vitamin D levels is also noted.^55^ In patients with SLE, a reduction in anti-dsDNA positivity is observed post-supplementation. Among those suffering from psoriasis, after 6 months of intervention, crude results of PASI score are improved.^57^ Recently, a large randomised study in the USA that investigated the incidence of autoimmune disease following vitamin D and marine n-3 fatty acids supplementation (VITAL study)^58^ and reported that ONS with vitamin D (2000 IU/day), with or without n-3 fatty acids, decreased the incidence of autoimmune disease (including RA, polymyalgia rheumatica and others) by 22% after 5 years of follow-up. The authors in this study raised questions whether the general recommendations for Vitamin D intake for the general population made by public health organisations, could be considered appropriate to achieve optimal levels for immune function. The mechanism of vitamin D on individual autoimmune diseases has not been elucidated so far, thus pointing to a need for further research.

As for vitamin C, it consists of an antioxidant nutrient playing a pleiotropic function in metabolism and health. Recent meta-analyses of RCTs indicate that ONS can improve gout outcomes, by inducing a significant reduction in serum uric acid (SUA) levels SUA,^59^ with younger patients benefiting the most. Moreover, ONS with vitamin C via peroral and intravenous routes can also reduce inteleukine-6 (IL-6) levels, improving the level of inflammation.^60^

Irrespective of disease status, micronutrient deficiencies have the potential to increase immunosenescence, fuelling up inflammation and susceptibility to infection.^61^ For example, folic acid deficiency is associated with inversely high homocysteine (HCY) levels, given that folate is a cofactor for methionine synthase, which in turn, catalyses the HCY conversion.^62^ In patients with RDs on methotrexate (MTX) therapy, folic acid concentrations are reduced, as a result of folate antagonism.^63^ To correct this, ONS with folate is common practice in parallel to the prescription of MTX.^64^ According to meta-analyses, folic acid supplementation can reduce the frequency of gastrointestinal side-effects and hepatotoxicity, lower circulating levels of transaminases and the rate of patient withdrawal from MTX treatment.^65^ Due to the low serum levels of folic acid, several meta-analyses of case-control studies also reveal higher circulating levels of HCY in patients with AS on MTX compared to those on anti-tumour necrosis factor α (anti-TNFα) treatment, indicating a greater CVD risk, irrespective of the methylenetetrahydrofolate reductase (*MTHFR*) C677T genotype.^65^ HCY acts in lowering the activity of the asymmetric dimethylarginine (ADMA) decomposing enzyme and reducing carotid intima-media thickness, leading to atherosclerosis and atheromatosis.^66^ In this manner, ONS with folic acid can effectively lower the concentration of serum HCY, reducing the incidence of stroke.^67^ Although folic acid does not belong to the classical immunonutrients, it appears that for rheumatology and cardiology, its importance in reducing cardiovascular risk and improving immune status is pivotal.^68^ As a result, greater levels of folic acid in patients with RA have been associated with a lower mortality risk.^69^

Last, but not least, vitamin E has anti-inflammatory and antioxidant potential, protecting against CVD. ONS with high doses of vitamin E can reduce the release rate of pro-inflammatory molecules (interleukine-8, plasminogen-activator inhibitor 1, TNF-α and CRP), lower lipid peroxidation and superoxide production, and decrease the rate of atheromatosis.^70–72^ Patients with a diagnosis of inflammatory skin diseases in particular, like psoriasis, were shown to have low serum vitamin E levels.^73^ Nevertheless, studies examining vitamin E ONS in RDs have only used animal models to date.

### Selenium and Zinc

Selenium (Se) and zinc (Zn) are important cofactors for the activity of antioxidant enzymes, regulate different aspects of inflammatory and immune responses and have emerged as two important micronutrients in RDs in recent years. They strengthen the antioxidant defence mechanisms and play pivotal roles in the regulation of innate and adaptive immune responses.^74–76^ Patients with SSc and psoriatic arthritis (PsA) tend to exhibit low serum Se levels,^77–79^ and although treatment does not appear to affect Se concentrations in RDs, an inverse relation has been noted with CRP and ESR concentrations.^80^ Moreover, in SSc, Se deficiency has been implicated in the progression of tissue fibrosis.^78^ Ma and colleagues^74^ pooled the data of patients with RA and revealed that they also exhibited low serum Se levels and Zn concentrations and increased copper (Cu) levels compared to healthy controls, suggesting that these micronutrients have potential roles in the pathogenesis of RA. Although oral supplementation with either Se or Zn may improve the clinical manifestations and disease activity of RA patients,^74^ there is limited evidence to support their routine supplementation in RA patients. Regarding Se, there is still debate whether the reason for the decreased Se concentrations in RA is the result of malnutrition associated with chronic conditions or is due to other reasons.^81^ The review by Qamar et al.81 highlighted the therapeutic potential of Se as an antioxidant in treatment of RA by exerting immunostimulant effects to enhance the functioning of immune cells and thyrocytes. However, the same authors expressed their concerns regarding the potential toxicity of Se supplementation in view of the narrow margin between beneficial and harmful intakes of Se.

Similarly to Se, both Zn deficiency and excess generate redox stress. It is also unknown if the reduced levels of Zn in RA patients is a consequence of an acute phase response in inflammation that removes Zn from the circulation or if it is a consequence of altered availability of Zn in tissues involved in the autoimmune aspects and pathogenesis of the disease.^76^ Moreover, severe Zn deficiency has been associated with decreased ratio of T helper cell to cytotoxic T cell, increased monocytes cytotoxicity and reduced natural killer cell activity.^74^

Different Zn preparations have been used in clinical trials with mixed outcomes.^76^ Interestingly, preliminary research on zinc oxide nanoparticles using a suitable animal model has shown promising beneficial effects by reducing inflammation and ameliorating autoimmunity.^75^

In this experimental study, the reductions of serum levels of cytokines were comparable to a standard drug (diclofenac) used in RA. Its safety and efficacy, however, is yet to be proven in humans. In addition, animal studies have indicated the therapeutic potential of Zn in other autoimmune disorders too such as multiple sclerosis (MS).^82,83^ Since autoimmune conditions are frequently associated with depression, the potential of Zn supplementation to reduce depressive symptoms - as was observed in a clinical trial with MS patients (220mg zinc sulphate containing 50 mg zinc element)^84^ - is worth of further investigation. More research and high-quality human intervention trials are needed to confirm the efficacy of Zn supplementation and establish the most optimal form, route of administration and dose levels in patients with RA and other autoimmune conditions. While Se and Zn currently have no place in the traditional treatment of RDs in academic medicine, restoration to optimal levels of these immunonutrients may provide significant opportunities as adjuvant therapy of some RDs such as RA and improve quality of life. Se deficiency may induce higher levels of inflammatory cytokines and impartial immune response, leading to increased susceptibility to infection.

### N-3 fatty acids

A plethora of studies have highlighted the anti-inflammatory action of n-3 fatty acids.^85^ They can modulate pro-inflammatory cytokine secretion and also participate in the restoration of homeostasis of tissues post-inflammation through the release of pro-resolving mediators.^86–88^ In RA, ONS with n-3 fatty acids ameliorates disease activity and leukotriene B4 concentrations.^89^ Furthermore, supplementation benefits extend far beyond rheumatology-related outcomes, improving the cardiovascular risk of patients with RA as noted by a reduction in blood triacylglycerol effects are to be seen in RDs, particularly in light of the non-clinically significant effects seen at low doses. However, these calls for an urgent need of risk-benefit assessment since systematic reviews^90^ have indicated that doses even greater than 5 g/day for a duration of 12 months may be required to show efficacy and provide the data to evaluate possible health risks.

### Probiotics

Probiotics, and in particular tolerogenic probiotics have been suggested as important modulators of disease activity in RDs.^91^ It appears that gut dysbiosis leading to disturbed interstitial permeability, molecular mimicry, and post-translational modifications, is a contributing factor in the development of RDs, including rheumatoid arthritis (RA), Sjögren’s syndrome (SS), systemic lupus erythematosus (SLE), systemic sclerosis (SSc) and ankylosing spondylitis (AS), through inducing a shift in the equilibrium between pro- and anti-inflammatory status and immune homeostasis.^92^ A recent study^93^ suggested that *Klebsiella* and *Escherichia* which can lead to enhanced intestinal permeability and inflammation, were more abundant in patients with RA compared to healthy adults. Furthermore, it is known that a “healthy” gut releases food metabolites such as short-chain fatty acids (SCFA)^94^ which are beneficial to maintain the integrity of the intestinal mucosa and anti-inflammation.^93^

For example, *Megamonas* which has been reported to participate in the metabolism of carbohydrates into SCFAs, is decreased in RA patients.^95^ Despite the fact that a number of studies have pointed to alterations in intestinal microbiota metabolites in RDs, according to Manasson,^96^ the research on microbiome has not yet reached its full potential in directing clinical practice. In patients with RDs, meta-analyses of randomized controlled trials (RCTs) indicate that oral nutrient supplementation (ONS) with probiotics resulted in an improvement in c-reactive protein (CRP) levels^97^ -with combined *Bifidobacteriales* and *Lactobacillales* formulations inducing the greatest response^98^-, an improved quality of life (*Lactobacillales*-only formulations),^98^ and a reduction in pain.^98^ On the other hand, as far as patients with psoriasis are concerned,^99^ probiotics ONS appears to improve psoriasis area and severity index (PASI) score. Nevertheless, research on the ONS with probiotics has not yet achieved its full potential.^96^

### Curcumin

Curcumin is a natural polyphenol and the main component of the turmeric plant (*Curcuma longa*), frequently consumed by patients with autoimmune diseases.^100^ ONS with curcumin has the potential to improve redox status of populations, by improving total antioxidant capacity (TAC) and decreasing decrease malondialdehyde (MDA) concentrations.^101^ However, curcumin is unstable at physiological pH, has low solubility in water, and is rapidly metabolised;^102^ hence, these parameters should be carefully taken into consideration when evaluating different preparations of curcumin and their efficacy in clinical trials. A recent systematic review indicated that in RA, ONS with curcumin (doses of 250–1500 mg/day) over 8–12 weeks is associated with a reduction in ESR and decreases in CRP concentrations.^103^ When a greater duration of supplementation (> 8 weeks) is adopted, paired with a large curcumin dosage (> 500 mg), the observed reductions in ESR and CRP are more profound.^103^ In psoriasis, all turmeric formulations appear to reduce symptoms.^104^ However, more human studies are required to confirm the efficacy and determine optimal preparations and doses of curcumin on RDs.

## CONCLUSIONS

The current evidence highlights the importance of immunonutrition in rheumatology and the need for educating resident rheumatologists on the proper use of immunonutrients. With inflammation, impaired immunity, increased risk of infection, musculoskeletal disorders and autoimmune atherosclerosis being in the core of RD’s pathophysiology, it appears that the manipulation of immunonutrients for achieving and attaining remission and health of patients gains further importance. Nevertheless, more high-quality research is required to help us understand the background mechanisms of action of immunonutrients in rheumatology, determine their effective and optimal doses and hence, guide clinical practice in an evidence-based manner.
